# Propensity-score matched comparison between minimally invasive and conventional aortic valve replacement

**DOI:** 10.3325/cmj.2022.63.423

**Published:** 2022-10

**Authors:** Hrvoje Gašparović, Petra Čerina, Tomislav Tokić, Marjan Urlić, Branka Golubić Ćepulić, Tomislav Kopjar, Ivan Burcar, Bojan Biočina

**Affiliations:** 1 Department of Cardiac Surgery, University Hospital Center Zagreb, Zagreb, Croatia; 2 Department of Transfusion Medicine and Transplantation Biology, University Hospital Center Zagreb, Zagreb, Croatia

## Abstract

**Aim:**

To evaluate the impact of minimally invasive aortic valve replacement (mini-AVR) on clinical outcomes in comparison with the gold standard.

**Methods:**

We retrospectively reviewed the records of all patients who underwent isolated AVR at the University Hospital Center Zagreb from 2010 to 2020. Patients undergoing mini-AVR were compared with patients undergoing conventional AVR (fs-AVR). The primary outcome measure was blood product consumption. Propensity score matching was used to create a balanced covariate distribution across treatment groups. Additionally, we compared the contemporary outcomes with a historical control.

**Results:**

The final sample consisted of 1088 patients. In the unmatched cohorts, mini-AVR patients were younger (65 ± 12 vs 68 ± 10 years, *P* < 0.001) and had lower risk profiles (EuroSCORE2 2.8 ± 2.0 vs 3.5 ± 3.1, *P* = 0.003). After matching, mini-AVR patients required less blood transfusion than fs-AVR patients (270 [0-790] vs 510 [0-970] mL, *P* = 0.029). The incidences of stroke, dialysis, new AV block, and mortality were comparable. Cross-clamp times were longer in the mini-AVR group (71 [60-87] vs 66 [53-83] minutes, *P* = 0.013). Outcomes were improved in the contemporary mini-AVR era compared with our early mini-AVR experience across multiple metrics. Blood product consumption was reduced in the latter tercile of experience (0 [0-520] vs 500 [0-1018] mL, *P* < 0.001), and the operation was performed more expeditiously (cross-clamp times: 63 [54,80] vs 74 [62,88] minutes, *P* < 0.001) in comparison with earlier periods.

**Conclusions:**

We showed that mini-AVR was associated with less blood product requirement than conventional surgery. Our data supports wider adoption of minimally invasive techniques in dedicated centers of excellence.

Aortic valve disease is the most prevalent cardiac valve disorder leading to hospital admission and cardiac care (1). No pharmacological treatment strategy is effective against progression of aortic valve stenosis (AS) or subsequent adverse left ventricular remodeling (1). The most common underlying etiology is calcific AS affecting either tricuspid or bicuspid aortic valves. Patients with bicuspid aortic valves tend to develop aortic valve disease earlier. Far less commonly, rheumatic pathology may play a role (1). The preclinical stage of AS is termed aortic sclerosis, and its prevalence is strongly related to patient age (1). Aortic valve disease is a mechanical problem, requiring a mechanical solution. Untreated symptomatic aortic valve stenosis carries a one-year mortality burden of nearly 25% (2). Aortic valve replacement is one of the safest and most efficient procedures in cardiac surgical practice. The conventional surgical procedure requires a full median sternotomy. Novel transcatheter approaches, however, have shifted the landscape of contemporary aortic valve disease treatment and have effectively challenged the conventional surgical approach. While transcatheter approaches were initially designed for patients whose operative risks were deemed prohibitive, they have become more prevalent among less morbid patients (3). This trend is likely to continue. Surgical aortic valve replacement, however, remains the standard of care for most patients (4). Additionally, surgery caters to patients who require both tissue and mechanical valve prosthesis. There are currently no alternatives to surgery for patients requiring mechanical valve prostheses. Notwithstanding the unquestionable efficacy of conventional full-sternotomy aortic valve replacement (fs-AVR), there is an acute need for wider dissemination of less invasive approaches in the cardiac surgical arena. Benefits of minimally invasive aortic valve replacement (mini-AVR) are both cosmetic and functional in nature (5). The former shape patient referral policies and profoundly affect the contemporary practice. The latter likely stem from partial preservation of anterior thoracic wall integrity and are manifested by earlier resumption of daily activities, reduction of pain, and lower transfusion requirements (5). In comparison with conventional AVR, minimally invasive procedures reduce operative trauma, while not exposing the patient to transcatheter aortic valve implantation-related risks of paravalvular leak, increased rate of pacemaker implantations, and vascular complications (6). Notwithstanding the potential benefits of mini-AVR, these have not been uniformly shown in all studies evaluating the impact of less invasive procedures (7,8). The benefits of minimally invasive approaches have previously been contested due to increased operative times (7,8). Technical challenges include less room for operative manipulation, more difficult de-airing, and need for conversion (4,8). Wider adoption of rapid deployment prostheses harbors potential to further accelerate implementation of mini-AVR into the standard of care for patients with aortic valve pathology. The aim of this study was to evaluate the impact of minimally invasive aortic valve replacement (mini-AVR) on clinical outcomes in comparison with the gold standard.

## METHODS

### Study design

**Figure 1:**
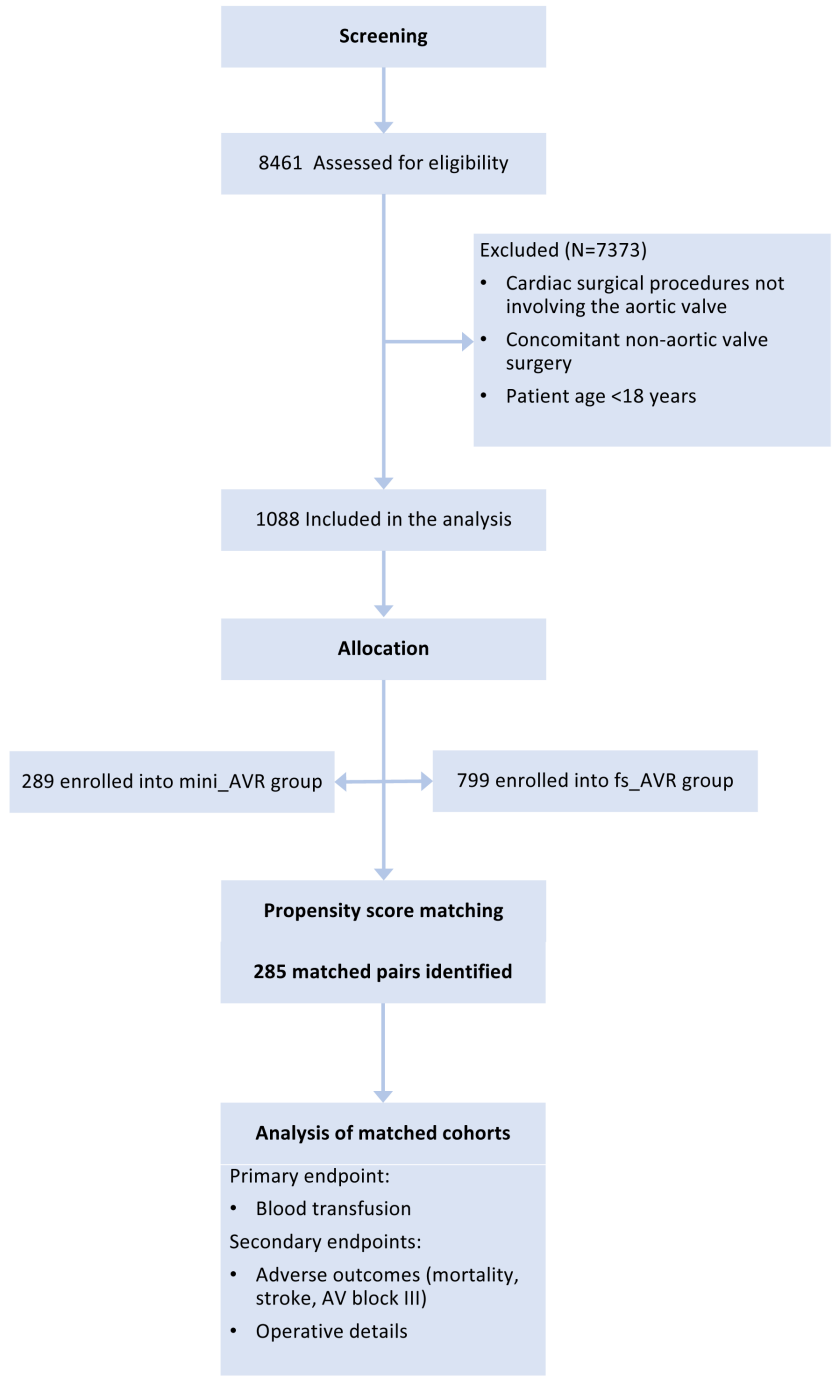
Study design flowchart detailing patient enrollment, allocation, and analysis.

From January 1, 2010 to December 31, 2020, the records of all patients in the University Hospital Center Zagreb’s cardiac surgical electronic database were screened for inclusion into this retrospective analysis. This digital search was complemented with manual record review to ensure completeness of data. The only inclusion criterion was first-time isolated aortic valve replacement. The exclusion criteria were age less than 18 years, reoperations, concomitant coronary, mitral or tricuspid valve surgery. Additionally, we excluded patients with ascending aortic surgery at the time of AVR, aortic valve repair, or full aortic root replacement. Preoperative mechanical circulatory support for hemodynamic instability was also an exclusion criterion. During the study period, 8461 cardiac surgical procedures were performed at our tertiary academic center. After the application of inclusion and exclusion criteria, 1088 patients remained in the final sample (Figure 1). The Institutional Review Board of the University Hospital Center Zagreb approved the study. Written informed consent was waived due to the retrospective nature of the study.

Individual medical records were reviewed for demographic, clinical, laboratory, and transfusion requirement data. The primary outcome measure was blood product utilization across the study and control populations. Additionally, we compared the contemporary outcomes with a historical control. The mini-AVR surgical experience was divided into terciles. The contemporary cohort consisted of patients operated on in the last tercile of our experience, while the historical control included patients operated on in the first two terciles of our experience.

### Surgical techniques

The conventional approach to the aortic valve is via a full midline sternotomy, while mini-AVR is performed via a limited sternotomy with a “J” extension into the third or fourth intercostal space. The surgical incisions for mini-AVR vary in length from 4.5 to 6 cm. Preoperative CT planning may assist in the selection of the appropriate intercostal space extension. We use central cardiopulmonary bypass (CPB) cannulation for both mini-AVR and fs-AVR. Adequate visualization is paramount for mini-AVR, and care must be taken not to compromise quality for less invasiveness. Particular care must, therefore, be paid to organizing the operative field in a way that facilitates aortic valve exposure. Vacuum assisted venous drainage is used ubiquitously, and flooding the operative field with CO_2_ facilitates later de-airing. The valve is exposed via similar aortotomies in both types of procedures, and there is no deviation from the standard in myocardial protection. Of note, we prefer a single-shot cardioplegia for all AVRs, which is especially useful for mini-AVR as it minimizes coronary ostial manipulation. The actual removal of the diseased valve, annular decalcification, and surgical replacement of the aortic valve are performed in the same way. Therein lies a major advantage of a limited sternotomy AVR over other types of minimally invasive procedures, as a similar skillset is needed to perform the central part of the procedure. Pacemaker wires need to be placed prior to aortic declamping, while the chest drainage tubes should be inserted with the heart decompressed on CPB. Additional procedures, such as ascending aortic replacement or enlargement of the aortic root for restrictive annuli, may be addressed via the minimally invasive approach.

### Statistical analysis

Continuous data are presented as mean values ± standard deviation or medians with interquartile ranges. Mann-Whitney U test was used for testing continuous data. Categorical variables and endpoints are presented as absolute numbers with percentages and were compared across groups by using 2 × 2 contingency tables. Measures of association were derived from the Fisher exact test. Propensity score methodology was used to reduce the confounding in statistical comparisons of outcomes of two treatment groups by accounting for differences in baseline patient characteristics. First, a logistic regression model was performed on preoperative patient characteristics (age, sex, EuroSCORE2, body mass index) to calculate the propensity score for each patient. Of note, EuroSCORE2 is a risk-assessment tool designed for cardiac surgical operations. It is based on both cardiac and patient-related factors. These include the presence of comorbidities such as chronic pulmonary disease, extracardiac arteriopathy, previous cardiac surgery, critical preoperative state, renal function and diabetes, as well as left ventricular ejection fraction, recent myocardial infarction, pulmonary hypertension, urgency of operation and functional status.

Calculated propensity scores represented the likelihood that the patient was in the mini-AVR treatment arm. Furthermore, a secondary analysis focusing on contemporary mini-AVR results was performed. First, the last tercile of our mini-AVR experience was compared with the first two terciles. Then, the contemporary mini-AVR cohort was compared with the contemporary fs-AVR cohort after propensity score matching. The data were analyzed with IBM SPSS Statistics, version 20.0 (IBM, Armonk, NY, USA).

## RESULTS

### Baseline patient characteristics

Of the 1088 patients in the final sample, 640 (59%) were men. The studied groups did not differ in sex distribution. Patients in the mini-AVR group were younger than patients undergoing fs-AVR (65 ± 12 vs 68 ± 10 years, *P* < 0.001). The incidence of hypertension was lower in the mini-AVR group (218 [75%] vs 659 [82%], *P* = 0.012), as was the estimated operative risk (EuroSCORE2 2.8 ± 2.0 vs 3.5 ± 3.1, *P* = 0.003). Creatinine clearance values were clinically similar in both groups (86 ± 33 vs 81 ± 35 mL/min, respectively, *P* = 0.009). Notwithstanding the significant difference, preoperative hemoglobin values were similar between the groups. The baseline characteristics of unmatched cohorts are summarized in Table 1.

**Table 1:** Baseline demographic and clinical profiles of the study population*

	Overall (n = 1088)	Full sternotomy AVR (n = 799)	Minimally invasive AVR (n = 289)	*P*
Age	67 ± 10	68 ± 10	65 ± 12	<0.001
Male sex, n (%)	640 (59)	467 (58)	173 (60)	0.727
Arterial hypertension, n (%)	877 (81)	659 (82)	218 (75)	0.012
Diabetes mellitus, n (%)	272 (25)	205 (26)	67 (23)	0.429
Hyperlipidemia, n (%)	565 (52)	409 (51)	156 (54)	0.450
Coronary artery disease^†^, n (%)	190 (17)	143 (18)	47 (16)	0.588
Smoking history, n (%)	239 (22)	169 (21)	70 (24)	0.282
Chronic obstructive pulmonary disease, n (%)	97 (9)	76 (10)	21 (7)	0.280
Atrial fibrillation/flutter, n (%)	181 (17)	145 (18)	36 (12)	0.027
EuroSCORE2	3.3 ± 2.9	3.5 ± 3.1	2.8 ± 2.0	0.003
Bicuspid AVR, n (%)	236 (22)	133 (17)	103 (36)	<0.001
Endocarditis, n (%)	32 (3)	22 (3)	10 (3)	0.545
Preoperative hemoglobin	132 ± 19	131 ± 19	135 ± 20	0.007
Body mass index, kg/m^2^	29 ± 5	29 ± 5	29 ± 5	0.147
Creatinine clearance, mL/min	82 ± 34	81 ± 35	86 ± 33	0.009
Left ventricular ejection fraction, %	56 ± 12	56 ± 12	57 ± 10	0.489

*Abbreviations: AVR – aortic valve replacement; EuroSCORE – European System for Cardiac Operative Risk Evaluation.

†Either non-obstructive coronary artery disease or previous successful percutaneous intervention.

### Propensity score matching

We performed propensity score matching to account for systematic differences in baseline characteristics between the two treatment groups. Two-hundred and eighty-five matched pairs were compared. Detailed patient characteristics of the matched cohorts are shown in Table 2. Conditional on the calculated propensity scores, the distribution of all baseline covariates was similar between the groups.

**Table 2:** Baseline demographic and clinical profiles of the propensity score matched cohorts*

	Full sternotomy AVR (n = 285)	Minimally invasive AVR (n = 285)	*P*
Age (years)	65 ± 11	65 ± 11	0.723
Male sex, n (%)	171 (60)	170 (60)	1.0
Arterial hypertension, n (%)	219 (77)	218 (76)	1.0
Diabetes mellitus, n (%)	61 (21)	67 (24)	0.616
Hyperlipidemia, n (%)	150 (53)	156 (55)	0.675
Coronary artery disease*, n (%)	39 (14)	47 (16)	0.413
Smoking history, n (%)	67 (24)	68 (24)	1.0
Chronic obstructive pulmonary disease, n (%)	26 (9)	21 (7)	0.543
Atrial fibrillation/flutter, n (%)	40 (14)	36 (13)	0.712
EuroSCORE2	2.77 ± 2.17	2.83 ± 2.05	0.445
Endocarditis, n (%)	8 (3)	10 (4)	0.812
Preoperative hemoglobin (g/L)	133 ± 19	134 ± 20	0.344
Body mass index, kg/m^2^	29 ± 5	29 ± 5	0.870
Creatinine clearance, mL/min	87 ± 40	86 ± 32	0.952
Left ventricular ejection fraction, %	56 ± 12	57 ± 10	0.648

*Abbreviations: AVR – aortic valve replacement; EuroSCORE – European System for Cardiac Operative Risk Evaluation.

### Perioperative outcomes in propensity score matched cohorts

**Figure 2:**
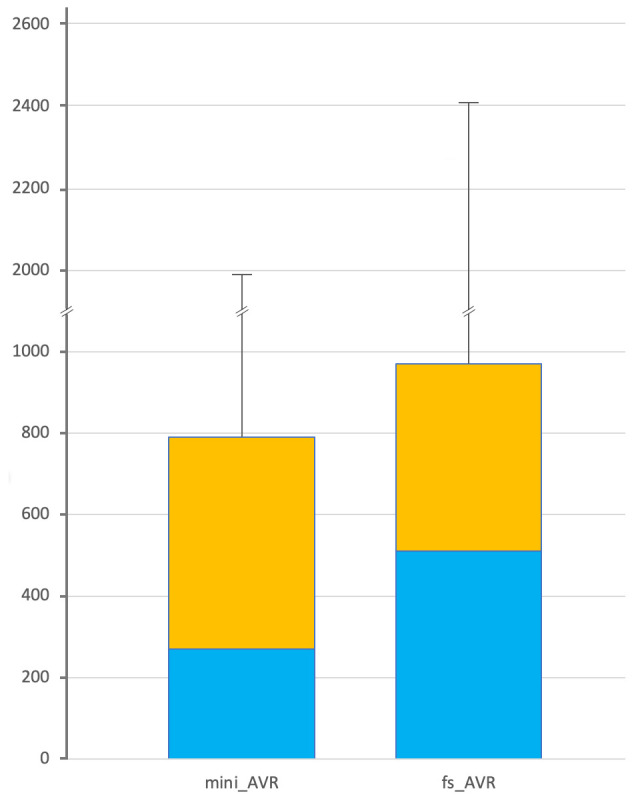
Reduced blood transfusion requirement among patients undergoing minimally invasive aortic valve replacement (mini-AVR) compared with patients undergoing full sternotomy (fs-AVR).

Analysis of the primary endpoint showed that patients in the mini-AVR group received less blood transfusions compared with their propensity-score matched controls undergoing fs-AVR (270 [0-790] vs 510 [0-970] mL, *P* = 0.029) (Figure 2). No differences in the volumes of transfused fresh frozen plasma (0 [0-758] vs 0 [0-760] mL, *P* = 0.520) or platelets (0 [0-0] vs 0 [0-0] units, *P* = 0.156) were observed. Perioperative outcome analysis is summarized in Table 3. Cross-clamp times and cardiopulmonary bypass times were longer in the mini-AVR group (71 [60-87] vs 66 [53-83] minutes, *P* = 0.013 and 102 [86-121] vs 96 [79-118] minutes, *P* = 0.026). While statistically significant, these differences bear little clinical relevancy. No difference in the duration of mechanical ventilation was observed for mini-AVR vs full sternotomy-AVR (7 [6-10] vs 8 [6-12] hours, *P* = 0.213), nor was there any clinically perceptible difference in the length of intensive care unit stay (2 [1-2] vs 2 [1-2] days, *P* = 0.045). The rates of adverse outcomes across the matched cohorts are detailed in Table 4. Patients in both groups shared similar incidences of postoperative renal replacement therapy, mechanical circulatory assistance, stroke, atrial fibrillation, or new pacemaker requirement. No difference in mortality was observed (5 [1.8%] in the mini-AVR group vs 6 [2.1%] in the matched full sternotomy group, *P* = 1.0).

**Table 3:** Surgical data and clinical outcomes in propensity matched cohorts of mini-AVR vs full sternotomy patients (all patients)*

Outcome	Median (IQR)	Mean rank	Sum of ranks	Test statistics	
**Blood component therapy (averaged per patient)**					
Packed red blood cells (mL)				Mann-Whitney U	36421.500
full sternotomy AVR	510 (0-970)	300.21	85558.50	Z	-2.177
minimally invasive AVR	270 (0-790)	270.79	77176.50	Effect size	0.0167
				P	0.029
Fresh frozen plasma (mL)				Mann-Whitney U	29559.500
full sternotomy AVR	0 (0-760)	244.55	64804.50	Z	-0.643
minimally invasive AVR	0 (0-758)	251.98	57955.50	Effect size	0.0015
				P	0.520
Platelets (units)				Mann-Whitney U	4417.500
full sternotomy AVR	0 (0-0)	96.50	9167.50	Z	-1.418
minimally invasive AVR	0 (0-0)	94.50	8977.50	Effect size	0.0071
				P	0.156
**Operative data**					
Myocardial ischemia (min)				Mann-Whitney U	35185.000
full sternotomy AVR	66 (53-83)	266.21	74806.00	Z	-2.498
minimally invasive AVR	71 (60-87)	300.54	85655.00	Effect size	0.0220
				P	0.013
CPB (min)				Mann-Whitney U	35698.000
full sternotomy AVR	96 (79-118)	268.04	75319.00	Z	-2.234
minimally invasive AVR	102 (86-121)	298.74	85142.00	Effect size	0.0176
				P	0.026
Valve prosthesis size (mm)				Mann-Whitney U	34631.500
full sternotomy AVR	23 (21-23)	290.76	81702.50	Z	-2.081
minimally invasive AVR	23 (21-25)	263.86	72032.50	Effect size	0.0152
				P	0.037
Intensive care unit (days)				Mann-Whitney U	36692.000
full sternotomy AVR	2 (1-2)	297.35	84149.00	Z	-2.003
minimally invasive AVR	2 (1-2)	271.74	77447.00	Effect size	0.0141
				P	0.045
Mechanical ventilation (h)				Mann-Whitney U	36558.000
full sternotomy AVR	8 (6-12)	287.97	80343.00	Z	-1.246
minimally invasive AVR	7 (6-10)	271.03	75618.00	Effect size	0.0055
				P	0.213

*Abbreviations: IQR – interquartile range; AVR – aortic valve replacement; CPB – cardiopulmonary bypass.

**Table 4:** Perioperative outcomes in propensity matched cohorts of mini-AVR vs full sternotomy patients (all patients)*

Perioperative outcomes	Full sternotomy AVR (n = 285)	Minimally invasive AVR (n = 285)	*P*
Reoperation for bleeding, n (%)	11 (3.9)	4 (1.4)	0.114
Sternal wound infection, n (%)	9 (3.2)	12 (4.2)	0.658
New dialysis, n (%)	5 (1.8)	5 (1.8)	1.0
New pacemaker, n (%)	2 (0.7)	7 (2)	0.176
New stroke, n (%)	1 (0.4)	2 (0.7)	1.0
Postoperative MCS, n (%)	1 (0.4)	1 (0.4)	1.0
Postoperative AF, n (%)	89 (31)	93 (33)	0.788
Mortality, n (%)	6 (2.1)	5 (1.8)	1.0

*Abbreviations: MCS – mechanical circulatory assistance; AF – atrial fibrillation.

### Contemporary outcomes vs historical control data

Acknowledging the inevitability of a multidisciplinary learning curve, we divided our surgical experience into terciles. We first compared the outcomes of mini-AVR patients operated on in the last tercile of our experience with those of patients operated on in the previous two terciles. Mini-AVR patients operated on in the last tercile required significantly less blood transfusions than their historical controls (0 [0-520] vs 500 [0-1018] mL, *P* < 0.001). Furthermore, the operation was performed more expeditiously, with myocardial ischemic times being significantly shorter in the last tercile (63 [54-80] vs 74 [62-88] minutes, *P* < 0.001). We also performed a propensity-score matched analysis of mini-AVR patients from the last tercile compared with patients undergoing fs-AVR during the same time frame. Ninety-five matched pairs were compared. The baseline preoperative characteristics are shown in Supplementary Table 1(Supplementary material 1). A clear trend toward lower blood product consumption was shown for the mini-AVR group (0 [0-520] vs 270 [0-750] mL, *P* = 0.066). Patients in the mini-AVR group had lower absolute mortality rates, although the difference did not reach significance (1 [1.1%] vs 4 [4.2%], *P* = 0.368). Detailed perioperative data and clinical outcomes are shown in Supplementary Table 2(Supplementary material 2) and Supplementary Table 3(Supplementary material 3).

## DISCUSSION

The current study is the largest single-center series of mini-AVR patients in Croatia. The study results confirm our hypothesis that minimally invasive approaches to aortic valve replacement can be performed safely and expeditiously in a large-volume tertiary academic center. Our data corroborate previous findings that emphasized the potential benefits of preserving a segment of the anterior thoracic wall. This has been hypothesized to translate into less postoperative pain, better mobility and earlier return to normal activities after discharge (7). The cosmetic advantage of mini-AVR is an important driver for patient referral to centers specializing in less invasive approaches. Benefits of limited sternotomy surgery have been challenged by potential drawbacks of less invasive approaches. These include more difficult de-airing of left-sided cardiac chambers, increased operative times, and compromised visualization.

Minimally invasive approaches have been repeatedly linked to increased durations of myocardial ischemia and cardiopulmonary bypass times (9). Our data challenges this notion as we demonstrated that, with increased experience in minimally invasive surgery, operative times can be reduced to levels on a par with conventional full sternotomy approaches. We strongly believe that dedicated minimally invasive teams are key to optimizing the outcomes of this subset of cardiac surgical procedures. Not only did we find that mini-AVR meets the quality and safety benchmarks set by conventional fs-AVR, but we also found that it offers quantifiable benefits. The primary outcome of our study was blood product consumption in relation to the surgical approach used for aortic valve replacement. In matched cohorts of patients, we showed that mini-AVR was associated with reduced blood transfusion requirement. Our data illustrate how the reduction in surgical trauma seen in mini-AVR could be part of a broader blood transfusion conservation strategy for patients with critical aortic valve disease. Benefits of mini-AVR, in terms of less bleeding, shorter ICU stay (10), and duration of mechanical ventilation, may be accentuated in obese patients (11).

The ultra-small incisions we use nowadays (≤5 cm in the most recent experience) are lasting visual representations of the limited extent of intrapericardial dissection, which translates into less surgical trauma. The superior cosmetic results are among the most important patient-driven factors for minimally invasive cardiac surgery. Body image and self-esteem metrics have been shown to be impaired after conventional cardiac surgery (12). In contrast, minimally invasive incisions have been shown to lead to psychological benefits and less anxiety symptoms due to superior esthetical wound healing and scar size (13). Another, albeit less frequently recognized, benefit of minimally invasive approaches in cardiac surgery is that future reoperations are less cumbersome and are associated with less bleeding. This observation stems from the fact that the caudal part of the sternum and the underlying pericardial sac remain intact during the primary, minimally invasive procedure, which allows for surgical entry into a seemingly virgin chest during reoperation. The expanding use of rapid deployment aortic valve prosthesis will likely enhance the adoption of minimally invasive strategies, as it simplifies the placement of a prosthesis and reduces all procedural times (6).

The present study is limited by its retrospective design and comprehensiveness of data input. A selection bias is difficult to avoid in this setting. This is underscored by the fact that blinding is impossible in the selection of the surgical approach. Mini-AVR is a more complex surgical procedure and is therefore more commonly performed by more experienced surgeons. Over 90% of these procedures were performed by a single surgeon specialized in minimally invasive approaches. In contrast, fs-AVR is performed by surgeons of variable surgical experience. Finally, associations observed in our study may be subject to unmeasured confounding.

In summary, factors driving the expedited adoption of minimally invasive aortic valve replacement are both patient driven and surgeon driven. We showed that the minimally invasive strategy to aortic valve replacement can be performed safely in experienced centers. Notwithstanding its more complex setup, we showed that mini-AVR is associated with lower blood transfusion requirement compared with conventional AVR.
