# Supplementary material for: Propensity-score matched comparison between minimally invasive and conventional aortic valve replacement
Source: Croat Med J. 2022 Oct;63(5):423–30. doi: 10.3325/cmj.2022.63.423 (PMC9648077; doi:10.3325/cmj.2022.63.423)
Supplement: Supplementary Table 1 [file CroatMedJ_63_s001.pdf]

Supplementary Table 1. Baseline demographic and clinical profiles of the contemporary propensity score matched cohorts (last tercile of surgical experience)

	**Full sternotomy AVR (n=95)**	**Minimally invasive AVR (n=95)**	***P*-value**
Age (years)	65±11	66±10	0.591
Male gender, n (%)	61 (64)	51 (54)	0.184
Arterial hypertension, n (%)	79 (83)	78 (82)	1.0
Diabetes mellitus, n (%)	27 (68)	25 (70)	0.871
Hyperlipidemia, n (%)	51 (44)	56 (39)	0.559
Coronary artery disease, n (%)	23 (72)	18 (77)	0.481
Smoking history, n (%)	36 (59)	29 (66)	0.359
COPD, n (%)	12 (13)	7 (7)	0.334
Atrial fibrillation/flutter, n (%)	13 (14)	11 (12)	0.828
EuroSCORE2	3.72±3.65	3.01±2.42	0.579
Endocarditis, n (%)	6 (6)	3 (3)	0.497
Preoperative hemoglobin (g/L)	131±19	138±20	0.029
Body mass index, kg/m^2	29±7	30±6	0.732
Creatinine clearance, mL/min	97±41	91±36	0.444
LVEF, %	56±11	56±8	0.362

AVR=aortic valve replacement; COPD=chronic obstructive pulmonary disease; EuroSCORE=European System for Cardiac Operative Risk Evaluation; LVEF=left ventricular ejection fraction
