# Supplementary material for: Propensity-score matched comparison between minimally invasive and conventional aortic valve replacement
Source: Croat Med J. 2022 Oct;63(5):423–30. doi: 10.3325/cmj.2022.63.423 (PMC9648077; doi:10.3325/cmj.2022.63.423)
Supplement: Supplementary Table 2 [file CroatMedJ_63_s002.pdf]

Supplementary Table 2. Surgical data and clinical outcomes in propensity matched cohorts of mini_AVR vs. Full sternotomy patients (latter tercile)

Outcome	Median (IQR)	Mean rank	Sum of ranks	Test statistics	
Blood component therapy (averaged per patient)					
Packed red blood cells (ml)				Mann-Whitney U	3858.000
Full sternotomy AVR	270 [0,750]	102.39	9727.00	Z	-1.838
Minimally invasive AVR	0 [0,520]	88.61	8418.00	Effect size	0,0119
				P	0.066
Fresh frozen plasma (ml)				Mann-Whitney U	1326.000
Full sternotomy AVR	510 [0,790]	53.79	3604.00	Z	-1.477
Minimally invasive AVR	720 [0,945]	62.79	2951.00	Effect size	0,0077
				P	0.140
Platelets (units)				Mann-Whitney U	564.000
Full sternotomy AVR	0 [0,0]	36.75	1764.00	Z	-0.361
Minimally invasive AVR	0 [0,0]	36.00	864.00	Effect size	0,0005
				P	0.718
Operative data					
Myocardial ischemia (min)				Mann-Whitney U	4077.000
Full sternotomy AVR	69 [52,84]	94.19	8383.00	Z	-0.417
Minimally invasive AVR	63 [54,80]	90.92	8637.00	Effect size	0,0006
				P	0.677
CPB (min)				Mann-Whitney U	4121.000
Full sternotomy AVR	97 [80,120]	93.70	8339.00	Z	-0.295
Minimally invasive AVR	94 [80,120]	91.38	8681.00	Effect size	0,0003
				P	0.768
Valve prosthesis size (mm)				Mann-Whitney U	3515.000
Full sternotomy AVR	23 [21,23]	93.37	8497.00	Z	-1.233
Minimally invasive AVR	23 [21,23]	84.37	7256.00	Effect size	0,0054
				P	0.217
Intensive care unit (days)				Mann-Whitney U	3976.500
Full sternotomy AVR	2 [1,2]	100.20	9418.50	Z	-1.438
Minimally invasive AVR	1 [1,2]	89.86	8536.50	Effect size	0,0073
				P	0.150
Mechanical ventilation (hrs)				Mann-Whitney U	4117.500
Full sternotomy AVR	7 [5,11]	97.66	9277.50	Z	-0.807
Minimally invasive AVR	7 [5,10]	91.27	8488.50	Effect size	0,0023
				P	0.420
