# Supplementary material for: Propensity-score matched comparison between minimally invasive and conventional aortic valve replacement
Source: Croat Med J. 2022 Oct;63(5):423–30. doi: 10.3325/cmj.2022.63.423 (PMC9648077; doi:10.3325/cmj.2022.63.423)
Supplement: Supplementary Table 3 [file CroatMedJ_63_s003.pdf]

Supplementary Table 3. Perioperative outcomes in the contemporary propensity matched cohorts of mini_AVR vs. Full sternotomy patients

Perioperative outcomes n (%)	Full sternotomy AVR (n=95)	Minimally invasive AVR (n=95)	*P*-value
Reoperation for bleeding	3 (3)	0 (0)	0.246
Sternal wound infection	5 (5)	3 (3)	0.721
New dialysis	0 (0)	1 (1)	1.0
New pacemaker	2 (2)	2 (2)	1.0
New stroke	0 (0)	1 (1)	1.0
Postoperative MCS	2 (2)	0 (0)	0.497
Postoperative AF	28 (67)	38 (40)	0.170
Mortality	4 (4.2)	1 (1.1)	0.368

MCS, mechanical circulatory assistance; AF, atrial fibrillation
